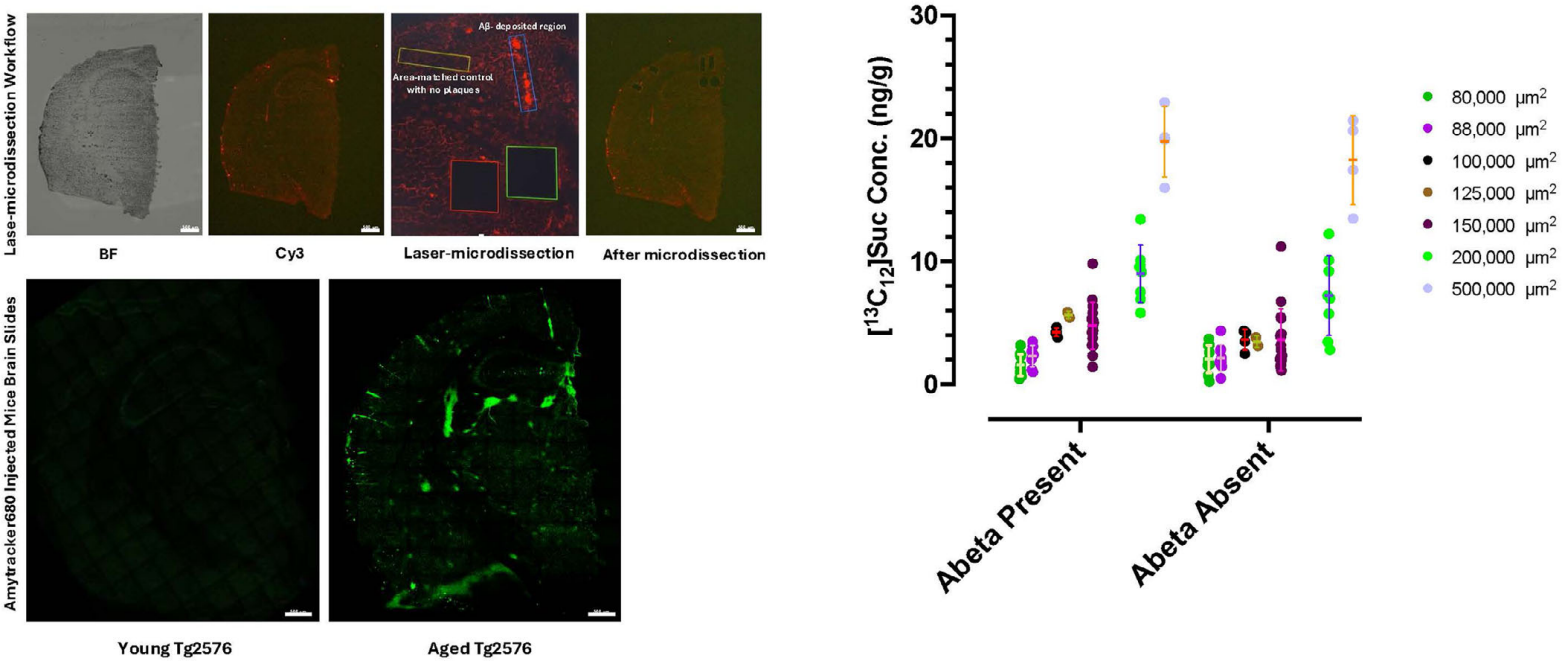# Assessing Blood‐Brain Barrier Integrity in Alzheimer’s Mouse Models: Implications for Therapeutic Delivery

**DOI:** 10.1002/alz70861_108470

**Published:** 2025-12-23

**Authors:** Ehsan Nozohouri, Behnam Noorani, Dhavalkumar Patel, Yeseul Ahn, Sumaih Zoubi, Ulrich Bickel

**Affiliations:** ^1^ Texas Tech University Health Sciences Center, Amarillo, TX USA; ^2^ Texas Tech University Health Sciences Center, Amarilllo, TX USA

## Abstract

**Background:**

Alzheimer’s disease (AD) is marked by extracellular amyloid‐beta (Aβ) plaques, intracellular tau‐containing neurofibrillary tangles, and cerebral amyloid angiopathy (CAA). While vascular amyloid deposition is a recognized driver of cerebrovascular dysfunction and blood‐brain barrier (BBB) disruption, the precise extent of BBB impairment in human AD and in transgenic mouse models of the disease remain unclear.

**Method:**

We assessed the BBB integrity at young and old age (6 and 16 months) in two mouse models, Tg2576 and 3Tg‐AD, and corresponding wild types. Under ketamine/xylazine anesthesia [^13^C_12_]sucrose, a small‐molecule marker of passive permeability, was injected as IV bolus. Blood samples were drawn up to 30 min. was injected at 29.5 min. Brain samples taken at 30 min immediately after injection of [^13^C_6_]sucrose as vascular marker and dissected into olfactory bulb, hippocampus, cortex and cerebellum. [^13^C_12_]sucrose and [^13^C_6_]sucrose concentrations were quantified by LC‐MS/MS. Brain uptake clearance K_in_ was calculated from brain concentrations, corrected for vascular space, and plasma AUC [^13^C_12_]sucrose. We applied mass spectrometry imaging by laser microdissection (LMD) of cryostat brain sections from aged Tg2576 mice to probe for local BBB integrity around vascular amyloid plaques identified by in vivo injection of Amytracker680. Quantitative immunofluorescence analysis using AIVIA software was performed for amyloid beta, tight junction proteins (claudin‐5, occludin, ZO‐1), tau protein and glial markers GFAP and IBA1.

**Result:**

Regional K_in_ values revealed no global BBB disruption in either young or aged Tg2576 and 3Tg‐AD mice compared to age‐matched wild‐type controls, even in presence of advanced Aβ pathology in the aged animals. Tight junction proteins showed stable expression across both models, except for local abnormalities and capillary morphological changes near Aβ plaques in aged Tg2576 mice. However, LC‐MS/MS of LMD samples did not reveal higher BBB permeability compared to samples without amyloid plaques, indicating functional preservation of the BBB in these areas (Figure 1).

**Conclusion:**

The BBB permeability remains largely intact in the Tg2576 and 3Tg AD models. Our findings highlight the requirement of effective drug delivery strategies for AD therapeutics. This study also supports the need for a multi‐method approach, incorporating both pharmacokinetic and imaging techniques, to thoroughly assess BBB integrity.